# Nrf2 Pathway in Huntington’s Disease (HD): What Is Its Role?

**DOI:** 10.3390/ijms232315272

**Published:** 2022-12-03

**Authors:** Paolo Tucci, Roberta Lattanzi, Cinzia Severini, Luciano Saso

**Affiliations:** 1 Department of Clinical and Experimental Medicine, University of Foggia, 71122 Foggia, Italy; 2 Department of Physiology and Pharmacology “Vittorio Erspamer”, Sapienza University of Rome, Piazzale Aldo Moro 5, 00185 Rome, Italy; 3 Institute of Biochemistry and Cell Biology (IBBC), National Research Council of Italy (CNR), Viale del Policlinico 155, 00161 Rome, Italy

**Keywords:** Nrf2, Huntington’s disease, neurodegenerative disease, oxidative stress, antioxidant

## Abstract

Huntington’s disease (HD) is an autosomal dominant neurodegenerative disease that occurs worldwide. Despite some progress in understanding the onset of HD, drugs that block or delay symptoms are still not available. In recent years, many treatments have been proposed; among them, nuclear transcriptional factor-2 (Nrf2) enhancer compounds have been proposed as potential therapeutic agents to treat HD. Nrf2 triggers an endogenous antioxidant pathway activated in different neurodegenerative disorders. Probably, the stimulation of Nrf2 during either the early phase or before HD symptoms’ onset, could slow or prevent striatum degeneration. In this review, we present the scientific literature supporting the role of Nrf2 in HD and the potential prophylactic and therapeutic role of this compound.

## 1. Introduction 

Huntington’s disease (HD) is an autosomal dominant neurodegenerative pathology that occurs worldwide and has an estimated prevalence of 2.71 per 100,000 individuals [1]. This number increases to 10.6–13.7 per 100,000 individuals in populations of European descent [2,3,4], whereas in Asian and South African countries, HD has a much lower prevalence (1–7 per million) [5]. Despite some progress in our understanding of HD’s etiology, none of the available drugs are able to block or delay symptoms. 

Currently, tetrabenazine is indicated to treat HD’s chorea symptom, an old drug that depletes cerebral monoamines through vesicular monoamine transporter type 2 inhibition [6,7]. Tetrabenazine is subject to variable CYP2D6 metabolism and often requires multiple daily administrations (three times a day) [8]. Recently, deutetrabenazine, a deuterate derivative of tetrabenazine, has shown better metabolism stability (longer half-life) and similar efficacy [9].

The role of oxidative stress (OS) in pathogenesis has been discussed in different studies conducted in preclinical HD models and in humans. Specifically, as the earliest events in HD, an increased OS linked to mitochondrial dysfunction and chronic inflammation has been shown. 

This review reports and discusses the scientific literature supporting the role in HD of the nuclear transcriptional factor-2 (Nrf2) that triggers an endogenous antioxidant pathway studied in different neurodegenerative disorders [10,11,12,13]. Moreover, a discussion on the possible prophylactic and/or therapeutic use of NRF2 activators in treating HD neurodegeneration will be presented.

## 2. HD Pathogenesis

The mean age for the onset of HD symptoms is 45 years, and usually, symptoms worsen as the disease progresses [14]. At the early stage, patients develop difficulty concentrating, memory lapses, depressive behavior, personality changes, and exaggerated and uncontrolled voluntary movements (chorea). At a later stage, daily activities are increasingly difficult and will require full-time nursing care [15]. These symptoms are a consequence of progressive degeneration in the brain of the striatum and cortex areas due to the aggregation of a mutant protein, mutant huntingtin protein (mHTT), that is transcribed instead of normal huntingtin protein (HTT) [6]. The mHTT is transcribed when the HTT gene shows an expansion of more than 35 repeats (in normal populations, repeats are between 9 and 35) of the nucleotide triplet cytosine-adenine-guanosine (CAG) coding for the amino acid glutamine. The high HD prevalence is linked to long CAG repeats [5], with HD being the most common of the nine polyglutamine diseases known [16].

In HD, it has been observed that the synthesis of toxic soluble monomeric protein mHTT is responsible for the formation of oligomers that are precursors of fibrils in the cell cytoplasm and nucleus [17,18,19]. This causes a loss of the HTT protective functions, such as its ability to counteract apoptotic factor activity and trigger Brain-Derived Neurotrophic Factor (BDNF) production [20,21,22]. 

The HTT also binds at and interacts with the DNA region of many genes, and the presence of an expanded polyglutamine tract in the protein results in transcriptional dysregulation [23,24].

**Figure 1:**
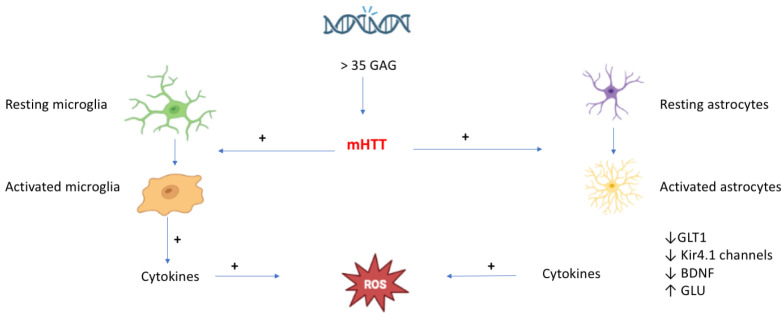
The consequence of the toxic protein mHTT synthesis in the HD (created in BioRender.com).

Moreover, two of the main protein degradation systems, the ubiquitin–proteasome (UPS) and autophagy ones, are both compromised in HD animal models as well as human tissue [25,26,27]. Based on the evidence available, the pathogenesis of HD requires a first step involving an abnormal repetition of CAG triplet leading to the production of mHTT, which in turn activates microglia and astrocytes. This activation triggers an intracellular pathway that induces altered gene expression and release of cytokines from microglia, along with a reduction of astrocytic glutamate transporter 1 (GLT1), Kir4.1 channels, BDNF, and an increase in both glutamate release and cytokines from astrocytes (with cytokines contributing to the production of OS and reactive oxygen species (ROS)) [15] (Figure 1).

## 3. ROS and HD

As mentioned above, there is evidence from human and animal models supporting the role played by OS in HD pathogenesis. 

It is well known that cellular physiological activity requires energy produced through a complex mechanism involving oxygen and mitochondria. In this context, the mHTT protein, by interacting with both outer and inner mitochondrial membranes, disrupts the import of mitochondrial proteins [28] and determines cell death [29]. 

A study reported that mitochondrial ultrastructure in a brain from a 20-year-old patient affected by juvenile HD was disrupted [30], while imaging studies showed that, in some brain regions, individuals with HD have lower levels of glucose metabolism and higher lactate concentration than healthy individuals [24,31].

The mHTT disrupts the anterograde and retrograde motility of mitochondria [32,33,34,35] and dysregulates mitochondrial biogenesis in cell and animal models of HD [36]. 

The role of the mitochondrial respiratory chain in HD has been confirmed in animals treated with 3-nitropropionic acid (3-NP), a compound that causes selective neuronal degeneration in the striatum and induces brain lesions similar to those observed in HD patients [37]. The 3-NP induced neurodegeneration involves mitochondrial membrane depolarization, energy depletion, oxidative stress, and enhanced mitochondrial-dependent apoptosis [38]. Therefore, 3-NP is largely used in animal models of HD.

The mitochondria are one of the main sources of ROS and reactive nitrogen species (RNS). In this organelle, O_2_ is reduced into superoxide (O^2−^) (that also originates from the NADPH oxidases (NOXs) pathway [39]) and hydroxyl radical (OH), both belonging to the ROS family. The ROS, along with hydrogen peroxide (H_2_O_2_), are produced in the cytoplasm by superoxide dismutase 1 (SOD1). In particular, H_2_O_2_ is also produced outside the cell by extracellular superoxide dismutase 3 (SOD3) and by cytochrome P450 during the β-oxidation of fatty acids. In the presence of Fe^2+^ or Cu^+^ (Fenton reaction), H_2_O_2_ is further transformed into hydroxyl radical (ROS) [40]. Other compounds belonging to the ROS family are the nitrogen species RNS such as peroxynitrite (ONOO^−)^, nitrogen dioxide radical (NO_2_), and nitryl cation (NO^2+^) [41].

The imbalance between ROS, RNS, and antioxidant molecules is responsible for OS production, with neurons and microglia largely involved in the process [42,43]. In the brain, the presence of unsaturated lipids, Fe^2+^ or Cu^+^, represents an ideal environment for neurotoxic lipid peroxidation [44,45] due to the modest antioxidant capacity of this organ.

In 1997, a study conducted in a postmortem HD brain reported a defect in mitochondrial energy metabolism and signs of DNA damage in the basal ganglia, and the same damage was found in both the striatum and cerebral cortex [46,47]. The marker for DNA oxidation, which leads to DNA fragmentation, was identified as an increased concentration of oxidized nucleotides, such as 8-hydroxy-2′-deoxyguanosine (8-OHdG) [48], with high levels of 8-OHdG detected in blood and post-mortem brain tissue of HD patients [49,50,51].

To better understand HD pathology, the R6/2 transgenic mouse model was developed. These mice express the human HD gene with the exon 1 presenting around 115 and 150 CAG repeats [52]; also, high levels of 8-OHdG were found in urine, blood, striatal DNA, and striatal microdialysates [53].

These data suggested the hypothesis that in HD, mitochondrial dysfunction leading to the overproduction of ROS is responsible for OS and nitrosative stress [54,55,56] with consequent neuronal dysfunction.

The damage caused by OS in HD seems to have two features: an early role in the neurodegeneration process and the induction of antioxidant and anti-inflammatory mechanisms [54,57,58]. 

The formation of peroxynitrite, a highly reactive product of nitric oxide and superoxide free radicals, is associated with striatal damage in HD animal models [59,60]. Moreover, high levels of 3-nitrotyrosine (3-NT), a marker of peroxynitrite formation [61], were observed in animal models [54,56] as well as in post-mortem HD brain tissue [49]. Furthermore, a reduction in plasmatic levels of glutathione [58] and dysregulation of lipid oxidation (increase in lipofuscin) were both detected in HD postmortem brain tissue [54,62,63]. 

A clear bi-directional interaction between mHTT and OS in HD was demonstrated in different studies [64]. The OS plays a critical role in the nuclear accumulation of mHTT [65], inducing proteasomal dysfunction, facilitating mHTT aggregation, and, finally, neuronal death [66]. The involvement of OS in CAG expansion was described in somatic and embryonic mouse cells [67,68].

Mitochondria are important in the synthesis of different neurotransmitters like dopamine (DA), norepinephrine, gamma-aminobutyric acid (GABA), serotonin, and glutamate [64]. This latter neurotransmitter seems to play an important role in HD pathogenesis by triggering excitotoxicity [69] associated with mitochondrial dysfunction [70]. Besides, glutamate excitotoxicity promotes OS [71] that counteracts cellular antioxidant mechanisms [72]. This mechanism worsens mitochondrial function, boosting neurodegeneration [73]. On the contrary, some evidence indicated that GABA could be involved in maintaining redox homeostasis and counteracting OS [74]. Regarding DA, the neurotransmitter (like other neurotransmitters with a catechol group), when metabolized by monoamine oxidases (MAO), contributes to both the generation of hydrogen peroxide (OS) [75] and the promotion of mHTT aggregate formation; a mechanism that can be reverted by the antioxidant ascorbate or by the selective c-Jun N-terminal kinase (JNK) inhibitor SP-600125 [76].

Based on results obtained in different studies, the use of antioxidant agents to reduce ROS accumulation was proposed as a therapeutic approach in HD. In this regard, two HD clinical trials evaluated the effect of creatine and coenzyme-Q10 on restoring mitochondrial function, but they failed to reach their endpoints [77,78].

The negative outcome of this approach has stimulated further research toward the finding of the potential of antioxidant stimulants with a potential therapeutic intervention for treating HD.

In this context, the cytosolic protein Nrf2, a member of the cap “n” collar proteins [79], has been described as playing a fundamental role in the antioxidant process in the brain.

**Figure 2:**
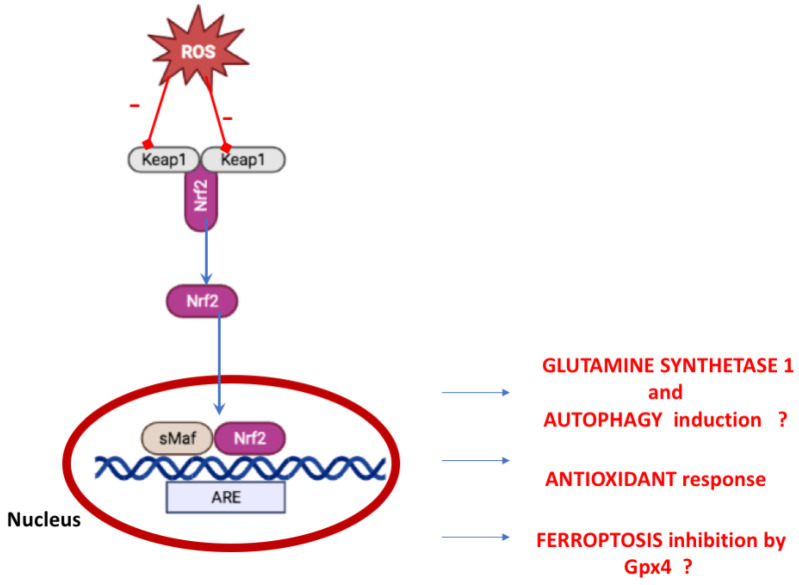
Keap1–Nrf2 Pathway. The oxidative stress leading to Nrf2 nuclear translocation and antixiodant transcription (created in BioRender.com). ? indicates no demonstrated mechanism in neurodegenerative disorders.

In physiological conditions, Nrf2 is inactive in the cytosol and bound to Kelch-like ECH-associated protein-1 (Keap-1) [80]. In case of physiological stress or OS, Nrf2 is dissociated from Keap1 and translocated into the nucleus, where it formed an heterodimer with musculoaponeurotic fibrosarcoma (Maf) protein (through the Neh1 domain), promoting the transactivation of the antioxidant response element (ARE) [81] (Figure 2). 

The Nrf2-ARE complex regulates the expression of cytochrome P450 oxidoreductases and phase II detoxifying enzymes and the expression of about 250 genes [82]. Other mechanisms independent of Keap1 have been proposed [83]. 

Nrf2 was shown to have an anti-inflammatory activity in the brain by counteracting neuroinflammation triggered by harmful stimuli related to neurodegenerative conditions [10]. Nrf2 is part of the formation of inflammasome, a multimeric protein complex that plays a role in inflammation. Specifically, Nrf2, when linked with the inflammasome sensor protein, nucleotide-binding oligomerization domain (NOD)-like receptor containing pyrin domain 3 (NLRP3), down-regulates inflammation [84]. 

Studies on cancer models (such as pancreatic tumors) showed a role of Nrf2 in glutamine and glucose metabolism [85,86]. These observations could also be suitable for the central nervous system considering that the glutamine is synthesized in astrocytes from the TCA cycle intermediate α-ketoglutarate arising from glucose metabolism. The glutamine synthesis is driven by glutamine Synthetase 1 (GS1), an enzyme ATP-dependent. GS1 contributes to glutamate homeostasis and is a critical piece in the glutamate–glutamine cycle, a crucial process between neurons and glia for the control of glutamate homeostasis [87]. It has been reported that the glutamate–glutamine cycle and GS1 are impaired in the HD post-mortem brain [88,89] in mice and Drosophila HD models [90,91,92]. Moreover, in neurons of this latter model, Gs1 gene (encoding for GS1) expression reduces the size of the toxic Htt-Q93 aggregates and increases the levels of autophagy [87]. In the fibroblasts of patients with HD, autophagy dysfunction has been reported due to the reduced levels of microtubule-associated protein 1A/1B-light chain 3(LC3) and GS1.

Moreover, Nrf2, by coworking with both BTB and CNC homology 1 (Bach1), a transcription factor member of the Cap ‘n’ Collar family [93], can stimulate or prevent gene expression involved in ferroptosis pathways [94]. Ferroptosis is a non-apoptotic, iron-dependent, pathway of programmed cell death, characterized by accumulating lipid peroxides playing a significant role in neurological disorders [95]. The inhibition of the ferroptosis pathway by Nrf2 has been reported in cancer cells, but direct evidence for the same activity in neurodegenerative disease is not reported. However, several features regulated by Nrf2 are involved in ferroptosis activation as the selenoenzyme glutathione peroxidase 4 (Gpx4). Gpx4 is localized in the brain and recognized as a suppressor of ferroptosis that need glutathione, whose genes are regulated by Nrf2 [96]. Hence, Nrf2 signaling could impact the ferroptosis activation in brain diseases.

## 4. Nrf2 and HD

In 2008, van Roon-Mom and coworkers [97] showed the involvement of Nrf2 genes in rat phaeochromocytoma PC12 cell lines, a cell model expressing exon 1 of the HD gene [98,99,100]. The authors, by inducing mHTT with doxycycline, found transcripts of Nrf2 responsive genes such as Nqo1, Gsta4, Gstp2, Gclc, Txnrd1, Me 1, thus confirming [101] the protective role of Nrf2 in the early stage of cellular pathology.

To further elucidate the role of Nfr2 in the HD model, triterpenoids (TPs), a class of chemical compounds able to potently induce the transcriptional activity of Nrf2 [102,103,104], were investigated. 

The 2-Cyano-3,12-Dioxooleana-1,9-Dien-28-Oic aci-methyl amide (CDDO-MA), a synthetic derivative of triterpenoid oleanolic acid with good penetration of the blood-brain barrier, was observed to exert neuroprotective effects against 3-NP neurotoxicity by inducing Nrf2 activation. These effects were abolished when Nrf2 was deactivated [105]. In another study, the effects of CDDO-ethyl amide (CDDO-EA) and CDDO-trifluoroethyl amide (CDDO-TFEA), CDDO derivatives showing higher brain concentrations compared to CDDO and CDDO-MA, were investigated in a N171-82Q transgenic mouse model of HD [106]. These transgenic mice express the 171 amino acids N-terminal fragment of human huntingtin containing 82 CAG repeats, and become HD symptomatic from 60 days of age and onward by exhibiting striatal atrophy [107,108]. The authors reported that CDDO-EA and CDDO-TFEA induced an increase in the transcription of genes regulated by Nrf2/ARE, a decrease in oxidative stress, improved motor performance, and increased survival and recovery of striatal atrophy. Flavonoids were also tested in their action against oxidative stress and inflammation induced by 3-NP neurodegeneration in male Wistar rats. Specifically, the naringin (4′,5,7-trihydroxy flavonone 7-rhamnoglucoside), the flavonone found in grapefruit and related citrus species, alleviates 3-NP-induced oxidative stress and inflammation through Nrf2 activation, which in turn increases the phase II/antioxidant genes [109]. Accordingly, a study investigating the naringin effects on 3-NP-induced neurotoxicity in pheochromocytoma cells (PC12 cells) showed a neuroprotective effect mediated by the Nrf2 activation of PI-3K/Akt-dependent pathway [110].

The effects of the flavone, luteolin, and its four synthetic derivatives Lut-C1, Lut-C4, Lut-C6, and Lut-C10) [111] were investigated in striatal cells derived from HD knock-in mice expressing mHTT (STHdhQ111/Q111) versus wild-type (STHdhQ7/Q7) [112]. The immortalized homozygote striatal cell lines, STHdhQ7/Q7 and STHdhQ111/Q111, arise from derivative striatal primordia of E14 mouse embryos expressing HTT with 7 polyQ and mHTT with 111 polyQ, respectively [113]. In the STHdhQ111/Q111 cells, higher numbers of fragmented mitochondria were detected compared to striatal cells expressing wild type HTT (STHdhQ7/Q7 cells) and impairment of Nrf2 signaling resistance to tert-buthylhydroquinone (tBHQ), a food preservative Nrf2 enhancer [113]. 

Moreover, STHdhQ111/Q111 cells showed a reduced expression of Nrf2 modulators Keap1 and p62 by activation of autophagy. The authors marked a limit of STHdhQ7/Q7 and STHdhQ111/Q111 cell models, expressing two copies of the mutant allele mHTT gene while HD is an autosomal dominant disease. 

Compounds Lut-C4 and Lut-C6, when tested at concentrations able to enhance cell viability, were observed to increase nuclear levels of Nrf2 and Nrf2/ARE transcriptional activity. Lut-C6 was also able to enhance SOD1 mRNA, SOD activity, glutamate-cysteine ligase catalytic subunit (GCLc) mRNA, and protein levels, whereas Lut-C4 was able to enhance GCLc mRNA levels only in mutant striatal cells. 

The role of Nrf2 in neuroprotection from 3-NP-induced neurotoxicity has been confirmed using cysteamine [114], that with its reduced form, cysteamine, inhibits several enzymes [115] and protects against neurodegeneration in different models of HD [116,117,118,119]. Oddly, when the YAC128 murine model of HD was treated with cysteamine, the absence of measurable plasmatic and cerebral levels of cysteamine and its metabolites was reported [120].

In a Phase I Dose Finding and Tolerability Clinical Study, cysteamine was tolerable in people with HD at a dose of 20 mg/kg per day [121]; moreover cysteamine is designated as orphan indication for HD (https://www.orpha.net/consor/cgi-bin/OC_Exp.php?lng=EN&Expert=24961, accessed on 27 October 2022) [121]. 

In cortical neural cell cultures, cysteamine activated the Nrf2 pathway in astrocytes while, following systemic administration in mice, the activation of the Nrf2 pathway was observed in the striatum [114].

The activity on astrocytes is shared with other Nrf2 activating molecules such as sulforaphane (SFN), a derivative of glucoraphanin found in *Brassica oleracea* [122], and tBHQ [123,124]. In animal models (C57BL/6 mice), daily administration of SFN (5.0 mg/kg/day, i.p.) 30 min before 3-NP treatment, showed preventive and therapeutic effects in the early stages of the 3-NP-neurotoxicity The SFN attenuated 3-NP-induced striatal toxicity by activating the Keap1-Nrf2-ARE pathway and inhibiting both MAPKs and NF-κB pathways [125].

Pre-treatment of 9 and 24-month-old Wistar female rats with tBHQ conferred a protective effect against 3-NP [126].

To test dimethylfumarate (DMF), an orally bioavailable fumaric acid ester that induces Nrf2, approved by the FDA to treat patients with relapsing forms of multiple sclerosis [127], the YAC128 mouse represents a suitable model. In this model, the human mHTT protein containing about 120 CAG repeats can also be expressed by conferring on the model similar characteristics to human HD disease, such as motor dysfunction, cognitive deficits, and age-dependent, progressive, and regionally selective neurodegeneration [128,129,130]. The DMF treatment exerted beneficial effects on survival time and motor functions in this model. This result was confirmed in the R6/2 mice models of HD, where DMF promoted the preservation of intact neurons and less pronounced dark cell degeneration in the striatum and motor cortex as well as up-regulation of the Nfr2 transcription factor in striatal neurons [130].

In *Panax ginseng*, a well-known traditional Chinese medicine, ginsenosides dammarane-type triterpene glycosides were isolated and classified into protopanaxadiol (PPD) or protopanaxatriol (PPT) types. The PPT is recognized as neuroprotective [131]. When administrated at 10 mg/kg dosage in a male Sprague-Dawley rat model of 3-NP-induced experimental HD, PPT increased Nrf2 entry to the nucleus, alleviated 3-NP-induced behavior disorders, reduced the overproduction of ROS, restored mitochondrial complex enzyme II and SOD activity, and directly scavenged superoxide anions and hydroxyl radicals [132].

Gintonin (GT), a lysophosphatidic acid receptor (LPARs) ligand found in ginseng [133], has been shown to regulate various cellular effects and to repress inflammation by exerting preventive and therapeutic effects in the early stages of striatal toxicity induced by 3-NP [134]. Interestingly, GT was observed to reduce cell death and mHTT aggregates in STHdh cells. These effects seem to involve different pathways such as LPARs, Nrf2, and MAPKs/NF-κB but disappear when Nrf2 is inhibited through siRNA or an inhibitor.

Based on the described results, many novel compounds able to activate Nrf2 were produced. In a study conducted in primary mouse cells and Drosophila models of HD, novel thiazole-containing inhibitors of deacetylase sirtuin-2 (SIRT2) with neuroprotective activity were discovered to possess an additional Nrf2-dependent mechanism [135]. In particular, a potent derivative MIND4-17 (lacking SIRT2 inhibitory activity) was identified as an Nrf2 activator. The activity of MIND4-17 was confirmed in mouse microglia and astrocytes and blood monocytes from patients with HD where the inflammatory response was blocked [136].

Fullerene C60 (an allotrope of carbon) neutralizes ROS and possesses a SOD-like activity [137], making it a suitable compound for preventing oxidative stress [138,139]. In 3-NP treated male rat Wistar model of HD, pre- and post-treatment with C60 prevented mitochondrial dysfunction and enhanced nuclear Nrf2 protein expression in the brain [140].

Different studies have shown a neuroprotective effect of curcumin (a natural polyphenol with multiple biological activities, including antioxidant and anti-inflammatory properties [141]) in experimental models of HD induced by 3-NP (reported in [142]), but the role of Nrf2 was not investigated.

In the rat model of 3-NP-induced HD, the administration of 2,4-diamino-6-hydroxypyrimidine (DAHP), previously showed to have a neuroprotective effect in cerebral ischemia [143]), stimulates the PI3K-AKT pathway and p-CREB leading to Nrf2 stimulation [144].

Diapocynin is an oxidative derivative of the naturally occurring agent apocynin, the most commonly used investigational NOX inhibitor [11]. Compared to apocynin, diapocynin is found to possess higher lipophilicity and greater potency to inhibit NOX activity [145,146]. Moreover, apocynin was found to attenuate motor alterations assessed as circling behavior and mitigate the striatal neuronal damage in an HD rat model induced by an intra-striatal injection of quinolinic acid [147]. Diapocynin alleviated 3-NP-induced behavioral and motor dysfunction in male Wistar rats, probably enhancing the Sirt1 pathway that, in turn, enhances Nrf2 expression with consequent inhibition of NOX2 (stimulation of oxidative stress) and NF-KBeta (stimulation of iNOS neuroinflammation), the stimulation of BDNF (inducing neuroprotection), GST, and GSH (inhibition of oxidative stress) [148]. 

Harmine, a plant-derived β-carboline alkaloid with a wide spectrum of pharmacological actions, including antioxidant properties [149], was shown to decrease intracellular aggregation of mHTT and OS in a yeast model of HD [150]. Furthermore, harmine increased the levels of Nrf2 protein as well as AMPK and p21, and restored redox homeostasis by attenuating 3-NP-induced neurodegenerative changes, as shown by improving rats ’motor and cognitive performance [151]. In Table 1, a summary of results from substances tested in vitro and/or in vivo is reported.

## 5. NRF2 Activators to Treat HD: Outlooks

**Figure 3:**
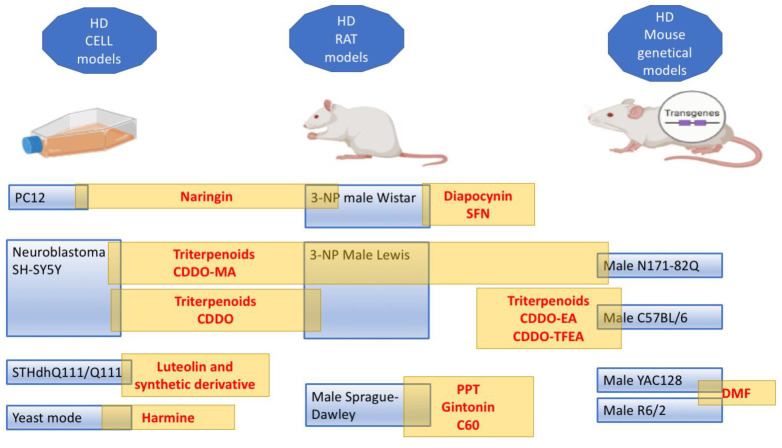
Evidence from substances treatment supporting a role for Nrf2 in HD. This figure reports a summary of results from substances tested in vitro and/or in vivo. The yellow rectangles reported substances active on the Nrf2 pathway in the different models (created in BioRender.com).

In this review, we reported the different agents studied to attempt to slow HD progression through Nrf2 pathway modulation. For this purpose, different in vitro and in vivo HD models were used. The preclinical studies must clarify some controversial data, such as the relationship between Nrf2, astrocytes, and neurons. First, it is to define whether the Nrf2 pathway is activated primarily in neurons or in astrocytes. If the Nrf2 pathway is central to astrocytes, it is unclear how the astrocytes rescue the neurons in the 3-NP-induced HD model. The neuroprotective effect of Nrf2 overexpression might not require Nrf2 delivery to neurons [132], and in such cases, the promotion of Nrf2 expression in astrocytes could be a worthwhile approach for protecting neurons [153]. This point of elucidation is fundamental considering the established role of astrocytes in the diseased brain, including HD [154]. Among the substances reported, only the TP derivative CDDO-MA was tested in cells, rat 3-NP HD models, and in the transgenic animal model of HD (N171-82Q mice) (Figure 3). Another TP derivative, CDDO, and the naringin were tested in cellular models (human neuroblastoma SH-SY5Y and PC12, respectively) and 3-NP HD model rats. Consequently, these studies provided strong evidence that these substances are potential candidates for clinical studies. Unfortunately, although promising, none of these agents were detected in clinical testing. The cysteamine was tested only in a dose-finding and tolerability trial. In the Introduction section, we reported the absence of drugs useful to prevent or arrest HD neurodegeneration. In recent years, many treatments have been proposed and rely on progress in genetic technique manipulation (for Therapies in the Pipeline see: https://hdsa.org/hd-research/therapies-in-pipeline/, accessed on 27 October 2022). Additionally, herbal products are tested as alternative strategies for treating HD. A phase II clinical trial was performed to evaluate the efficacy of cannabidiol and tetrahydrocannabinol, psychoactive components of *Cannabis sativa* L. (Cannabaceae), indicating that a high dose is required to overcome motor functions (https://www.clinicaltrials.gov/ct2/show/record/NCT01502046, accessed on 27 October 2022). Another, clinical trial is ongoing to establish the relationship between caffeine consumption and premanifest HD, considering the ability of caffeine to change striatal volume (https://www.clinicaltrials.gov/ct2/show/record/NCT03034122, accessed on 27 October 2022).These herbal products do not possess an activity on the Nrf2 pathway. In a clinical trial, the therapeutic potential of resveratrol was investigated on caudate volume in patients with HD (https://www.clinicaltrials.gov/ct2/show/study/NCT02336633?term=resveratrol&cond=Huntington+Disease&draw=2&rank=1, accessed on 27 October 2022). This clinical study stems from the neuroprotective effect of resveratrol observed in 3-NP-induced male C57BL/6J strain mice, 3-NP-treated rats, and N171-82Q transgenic mouse model of HD. Safety, tolerability, and cognitive improvement ability of EGCG, a green tea constituent, was estimated in a randomized double-blind phase II clinical trial in patients with HD and the expected outcomes would be an improvement in cognitive impairment (https://www.clinicaltrials.gov/ct2/show/record/NCT01357681, accessed on 27 October 2022). The results of the trial were not published. Resveratrol and EGCG were not considered in this review because the consequences of Nrf2 stimulation were not studied in HD animal models. However, these compounds showed cognitive enhancer activity in an animal model mediated by Nrf2 pathway stimulation [12]. Currently, DMF is the sole agent clinically available to treat neurodegenerative disease (relapsing-remitting multiple sclerosis) that acts through Nrf2 activation [155]. The goal of HD therapy should be directed toward the recovery of atrophy and loss of neuronal cell function. The activation of Nrf2, although in some studies induces the production of nerve growth factor as BDNF (like in the case of diapocynin and harmine), essentially mobilizes the expression of cytoprotective genes and the potential beneficial effects of NRF2 activators in the studies reported result from the prevention of neurotoxicity, neuroinflammation, and neurodegeneration. Hence, an ancillary potential role of compounds that act as Nrf2 enhancers in HD therapy clearly come out from studies discussed in this review. Probably, the stimulation of Nrf2 in an early phase of disease or before the symptom onset could slow or prevent the striatum degeneration. Overall, the prophylactic therapy in the HD is easy to perform because patients can know their risk of developing the disease by performing a diagnostic test revealing CAG repeats. Currently, the clinical trials are needed to attribute doses and timing of the treatments with Nrf2 modulators. Finally, it is noteworthy to note that only one, among the reported studies, used female animals although a cohort study (involving 67 millions of USA citizens) reported a significantly higher prevalence of HD in women (7.05 per 100,000, in men 6.91 per 100,000) [156]. Moreover, in the HD animal model, some gender differences were reported, suggesting the need to add gender as a biological variable in the research [157,158].

## 6. Literature Search Methods

In this review, we included peer-reviewed papers published in PubMed till 30 September 2022. Only peer-reviewed original research articles and reviews written in English were assessed for evaluation and inclusion in this manuscript.

## Figures and Tables

**Table 1:** Evidence from substance treatments supporting a role for NRF2 in HD models. In this table are reported a summary of results from substances tested in vitro and in vivo.

Compound	In Vitro Effects		In Vivo Effects	
CDDO-methyl amide (CDDO-MA)	Neuroprotective effects against 3-NP neurotoxicity in human neuroblastoma SH-SY5Y cells	[105]	Neuroprotective effects against 3-NP neurotoxicity in male C57BL/6 mice in male Lewis rats	[105]
CDDO-ethyl amide (CDDO-EA) CDDO-trifluoroethyl amide (CDDO-TFEA)			Improved motor performance, increased survival, and rescue of striatal atrophy in the N171-82Q transgenic male mice	[106]
Naringin	Neuroprotective effects against 3-NP neurotoxicity in pheochromocytoma cells PC12 cells	[110]	Alleviates 3-NP-induced oxidative stress and inflammation in male Wistar rats	[152,109]
Cysteamine			Neuroprotection against 3-NP neurotoxicity in male mice	[114]
Sulforaphane (SFN)			Preventive and therapeutic effects against 3-NP neurotoxicity in male C57BL/6 mice	[125]
Tert-buthylhydroquinone (tBHQ)			Protective effect against 3-NP in old female Wistar rats	[126]
Dimethylfumarate (DMF)			Beneficial effects on survival time and motor functions in male YAC128 mice. Preservation of intact neurons and less pronounced dark cell degeneration in the striatum and motor cortex in male R6/2 mice	[130]
Protopanaxatriol (PPT)			Alleviated 3-NP-induced behavior disorders in male Sprague-Dawley rats	[132]
Gintonin (GT)	Reduced cell death and mHTT aggregates in STHdh cells.	[134]	Preventive and therapeutic effects in the early stages of striatal toxicity by the 3-NP in male mice	[134]
Thiazole (MIND4-17)	Block of inflammatory response in mouse microglia, astrocytes, and blood monocytes from patients with HD	[136]		
Fullerene C60			Pre-treatment and post-treatment prevented mitochondrial dysfunction in 3-NP male Wistar rats	[140]
Diapocynin			Alleviated 3-NP-induced behavioral and motor dysfunction in male Wistar rats	[148]
Harmine	Decreased intracellular aggregation of mHTT in a yeast model of HD	[150]	Attenuated 3-NP-induced neurodegenerative changes and improving motor and cognitive performance in male Wistar rats	[151]
